# Bioactivity-Guided Isolation of Ethyl-*p*-methoxycinnamate, an Anti-inflammatory Constituent, from *Kaempferia galanga* L. Extracts

**DOI:** 10.3390/molecules17078720

**Published:** 2012-07-23

**Authors:** Muhammad Ihtisham Umar, Mohd Zaini Asmawi, Amirin Sadikun, Item J. Atangwho, Mun Fei Yam, Rabia Altaf, Ashfaq Ahmed

**Affiliations:** 1 Department of Pharmacology, School of Pharmaceutical Sciences, Universiti Sains Malaysia, Minden 11800, Penang, Malaysia; Email: amzaini@usm.my (M.Z.A.); aijustyno@yahoo.com (I.J.A.); yammunfei@gmail.com (M.F.Y.); rabia.ihtisham@gmail.com (R.A.); raza_chohan487@hotmail.com (A.A.); 2 Department of Pharmaceutical Chemistry, School of Pharmaceutical Sciences, Universiti Sains Malaysia, Minden 11800, Penang, Malaysia; Email: amirin@usm.my

**Keywords:** ethyl-*p*-methoxycinnamate, carrageenan-induced edema, cyclooxygenase, *Kaempferia galanga*, anti-inflammatory activity

## Abstract

This study evaluated the anti-inflammatory effect of *Kaempferia galanga *(KG) using an activity-guided approach. KG rhizomes were serially extracted with petroleum ether, chloroform, methanol and water. These extracts (2 g/kg each) were tested for their ability to inhibit carrageenan-induced rat paw edema. The chloroform extract was found to exert the highest inhibition (42.9%) compared to control (*p* < 0.001), hence it was further fractionated by washing serially with hexane, hexane-chloroform (1:1) and chloroform. The chloroform fraction (1 g/kg) showed the highest inhibitory effect (51.9%, *p* < 0.001) on carrageenan-induced edema. This chloroform fraction was further fractionated with hexane-chloroform (1:3) and chloroform, and of the two fractions, the hexane-chloroform sub-fraction was the most effective in inhibiting edema (53.7%, *p* < 0.001). GC-MS analysis of the active sub-fraction identified ethyl-*p*-methoxycinnamate (EPMC) as the major component, which was re-crystallized. EPMC dose-dependently inhibited carrageenan-induced edema with an MIC of 100 mg/kg. Moreover, in an *in vitro* study, EPMC non-selectively inhibited the activities of cyclooxygenases 1 and 2, with IC_50_ values of 1.12 µM and 0.83 µM respectively. These results validate the anti-inflammatory activity of KG which may be exerted by the inhibition of cyclooxygenases 1 and 2. EPMC isolated from this plant may be the active anti-inflammatory agent.

## 1. Introduction

Inflammation can be simply defined as a response of tissue to cell injury. It may be involved in almost every pathological condition that causes cell injury or necrosis. That is why inflammation is a common concern in a number of diseases, ranging from minor insect bites to much more complicated and serious conditions like cancer. It is newly been evidenced from serological studies of different biomarkers as well as by recently emerging imaging technologies that chronic vascular inflammation is involved not only in atherosclerosis, but also in other related conditions such as arterial hypertention and metabolic syndrome [1]. Chronic inflammation linked with various pathologies such as infectious diseases, cancer or autoimmune disorders result in a significant immunosuppression by inhibiting natural killer cells and T cells, thus aggravating the ailments [2]. This immunosuppression results from a number of factors, including activation of immune suppressor cells and pro-inflammatory cytokines that may impede the success of immune-based treatments. Prolonged inflammation may even lead to genomic instability, abnormal gene expression, and over secretion of pro-inflammatory mediators that pave the way for neoplasia [3,4]. Most of the conventionally used non-steroidal anti-inflammatory drugs (NSAIDs) have considerable gastrointestinal side effects. A long term treatment of a chronic inflammatory condition by using conventional NSAIDs is likely to cause clinically silent enteropathy with increased intestinal permeability that may cause intestinal inflammation [5]. Management of inflammatory conditions has always been a primary focus for pharmacologists, particularly when screening new phytoconstituents for their possible pharmacological tendencies that can be safer than conventionally available drugs. There is always a need to find newer constituents from traditionally used anti-inflammatory herbs that are safer than these conventional NSAIDs when used to treat chronic inflammatory disorders.

*Kaempferia galanga* Linn. (Zingiberaceae) is indigenous to tropical Asia, where it is commonly used in traditional medicine for the management of swelling, rheumatism, cough, dysentery, diarrhea, and stomachache [6,7]. In spite of these valuable medicinal properties, *K. galanga* is still comparatively little known and unutilized [8]. However, a number of investigations have been carried out to support these traditional claims, namely: the extracts of *K. galanga* are reported to exhibit nematicidal [9,10,11], mosquito repellent and larvicidal [12,13,14,15] activities. Other reported properties include sedative [16], anti-microbial [17,18], vasorelaxant [19,20,21], anti-neoplastic [22,23,24], anti-allergic [25], anti-oxidant [26,27], analgesic [28] and wound healing [29] effects. Ethyl-*p*-methoxycinnamate (EPMC) isolated from *K. galanga* extracts is found to be responsible for the pharmacological actions including, nematicidal, mosquito repellent, anti-neoplastic and anti-microbial effects [11,14,17,23,30], whereas ethyl cinnamate, a vital constituent of this plant, is said to be responsible for its vasorelaxant effects [19]. 

In an earlier biological activity screening study, the water extract of *K. galanga* was shown to exert a dose-dependent inhibition of induced rat paw edema [31]. More recently, the methanol extract of *K. galanga* was reported for its ability to dose dependently inhibit carrageenan induced hind paw edema and cotton pellet granuloma in rats [32]. However, adequate research on a medicinal plant should beyond screening for biological activity, aim at systematic standardization and develop into natural products or dosage forms which would effectively complement or supplement existing conventional therapies. To the best of our knowledge, such a systematic study of the anti-inflammatory activity of *K. galanga* has not been carried out, neither has any constituent in this plant been labeled with such activity. Consequently, the present study was designed to evaluate the anti-inflammatory effect of *K. galanga *using an activity-guided approach, with the aim to identify the constituent(s) responsible for the anti-inflammatory activity. 

## 2. Results and Discussion

### 2.1. Results

#### 2.1.1. Acute Toxicity Study

An acute toxicity study of crude extracts of *K. galanga* was conducted according to the Organization of Economic Co-operation Development (OECD) guideline 420, where the limit test dose of 5,000 mg/kg was used. No treatment-related mortality was observed at 5,000 mg/kg and throughout the 14 days observation period. In addition no significant changes such as apathy, hyperactivity, morbidity, *etc*. were observed in the behavior of the animals. No abnormal physiological changes attributable to treatment were noticed, including body weight, respiration rate and heart rate. Therefore *K. galanga* extracts were found to be safe at dose level of 5,000 mg/kg, and LD_50_ value was considered to be higher than 5,000 mg/kg.

A similar acute toxicity study of purified EPMC was also conducted later in the experiment according to OECD guideline 420 where a dose of 2,000 mg/kg was set as the limit test dose. EPMC was safe at the dose of 2,000 mg/kg and LD_50_ value of EPMC was found to be higher than 2,000 mg/kg.

#### 2.1.2. Preliminary Anti-inflammatory Effect of Crude Extracts of *K. Galanga*

**Figure 1:**
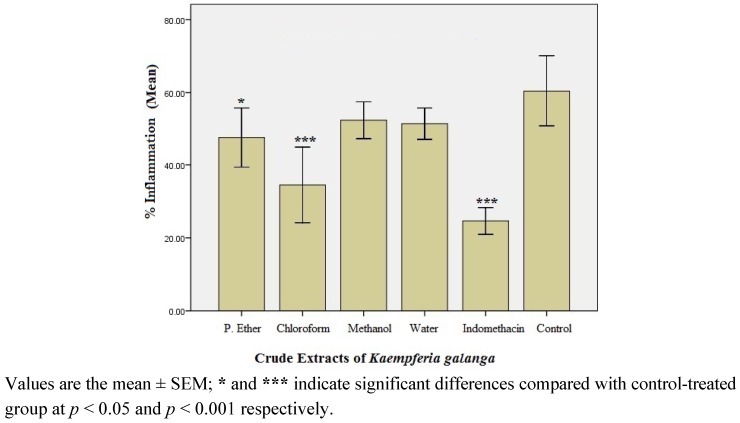
Percentage (%) inflammation observed in crude extract-treated rats after 3rd h of carrageenan administration (n = 6).

The percentage inflammation recorded in rats treated with crude *K. galanga* extracts-petroleum ether, chloroform, methanol and water extracts is presented in Figure 1. Of the four crude extracts, the chloroform extract showed the maximum anti-inflammatory effect (42.9% inhibition, *p* < 0.001) when compared with control; hence it was considered to be the most active crude extract.

#### 2.1.3. Anti-inflammatory Effect of the Fractions of the Active Chloroform Extract

**Figure 2:**
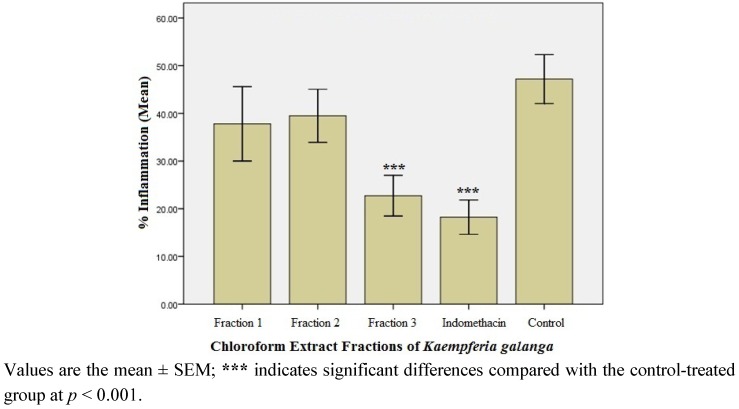
Percentage (%) inflammation observed in the fraction-treated rats after 3rd h of carrageenan administration (n = 6).

The active chloroform extract was subjected to liquid-liquid fractionation to give three fractions, *i.e*., fraction 1 (F-1), fraction 2 (F-2) and fraction 3 (F-3), which were evaluated for anti-inflammatory effect. Figure 2 shows the % inflammation recorded in the fraction-treated rats. F-3 was found to exert the highest anti-inflammatory activity, *i.e*., 51.9% inhibition of inflammation when compared to control (*p* < 0.001).

#### 2.1.4. Anti-inflammatory Effect of the Sub-fractions of Fraction 3

**Figure 3:**
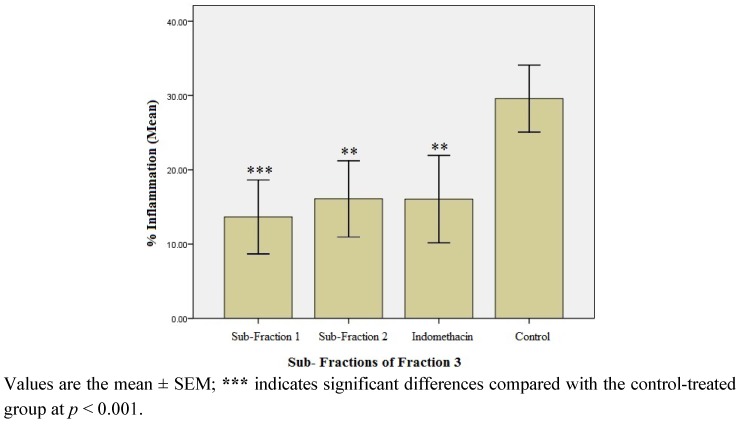
Percentage (%) inflammation observed in the sub-fraction-treated rats after 3rd h of carrageenan administration (n = 6).

F-3 was further fractionated to afford sub-fractions 1 (SF-1) and 2 (SF-2). Figure 3 shows the % inflammation recorded in rats treated with SF-1 and SF-2. Both sub-fractions significantly inhibited carrageenan-induced edema (*p *< 0.01). However, SF-1 showed maximum inhibitory effect (53.7% inhibition) when compared with the control (*p* < 0.001). The inhibitory effect of SF-1 was also found to be more potent than the standard anti-inflammatory drug, indomethacin (45.6% inhibition, *p* < 0.01).

#### 2.1.5. Gas Chromatography-Mass Spectrometry (GC-MS) Analysis of SF-1

**Figure 4:**
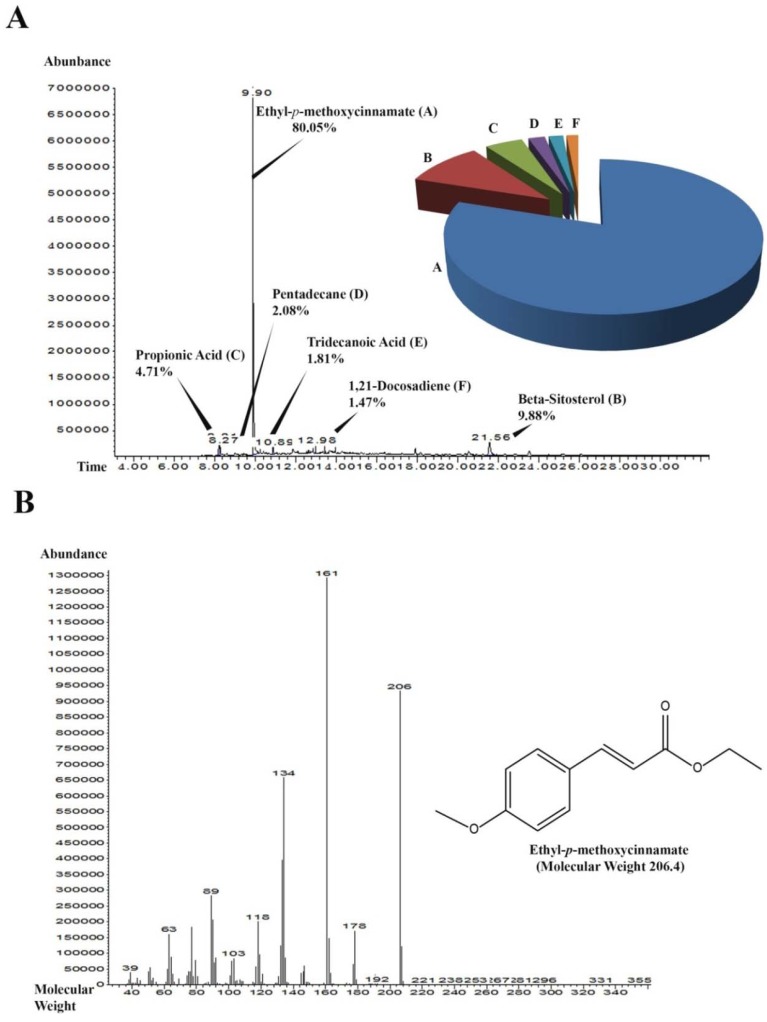
GC-MS chromatogram of the most effective sub-fraction (SF-1). (**A**) Peaks of the components detected in sub-fraction 1 along with their retention time. The pie chart represents the relative percentage abundance of each constituent detected. (**B**) Fragmentation of the most abundant peak with retention time 9.90. The most abundant compound was ethyl-*p*-methoxycinnamate (EPMC) with estimated molecular weight of 206.4.

Sub-fraction 1 (SF-1) was subjected to GC-MS analysis that showed the most abundant peak with retention time 9.90 min and 7,000,000 abundance to be ethyl-*p*-methoxycinnamate (EPMC) as shown in Figure 4. Fragmentation of the peak showed the molecular weight of the most abundant compound to be approximately 206.4. The second most abundant peak was β-sitosterol with a retention time 21.56 min and abundance of less than 500,000. Over all, SF-1 consisted of 80.05% EPMC, 9.88% was β-sitosterol, 4.71% propionic acid, 2.08% pentadecane, 1.81% tridecanoic acid and 1.47% 1,21-docosadiene.

#### 2.1.6. Isolation of Ethyl-*p*-methoxycinnamate (EPMC) Crystals

The crystals of EPMC were isolated from SF-1 and confirmed thereafter by proton nuclear magnetic resonance (Section 3.3.1).

#### 2.1.7. *In Vivo* Anti-inflammatory Effect of Ethyl-*p*-methoxycinnamate (EPMC)

**Figure 5:**
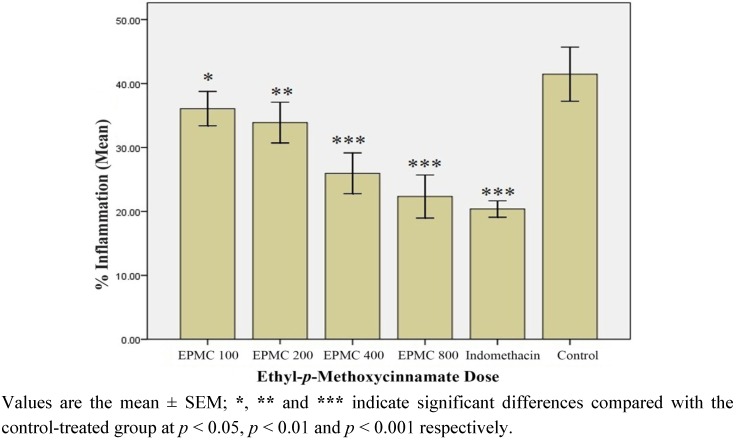
Percentage (%) inflammation observed in ethyl-*p*-methoxycinnamate (EPMC)-treated rats after 3rd h of carrageenan administration (n = 6). EPMC 100, EPMC 200, EPMC 400 and EPMC 800 indicate ethyl-*p*-methoxycinnamate in dose of 100 mg, 200 mg, 400 mg and 800 mg/kg.

Figure 5 represents the % inflammation recorded in rats treated with graded doses of pure EPMC. Although significant when compared with control (*p *< 0.05), the % inflammation recorded in the rats treated with 100 mg/kg of EPMC was apparently not very different from the control (13.3% inhibition), suggesting this dose to be the minimum inhibitory concentration (MIC); as a further decrease in dosage is likely to produce a non-significant effect. However, a successive increase in the dose of EPMC was seen to correspondingly produce a dose-dependent inhibition in rat paw edema (*p *< 0.01; *p *< 0.001).

#### 2.1.8. *In Vitro* Anti-inflammatory Effect of Ethyl-*p*-methoxycinnamate (EPMC)

In an *in vitro* assay, EPMC was found to inhibit cyclooxygenase enzymes 1 (COX-1) and 2 (COX-2) by 42.9% and 57.82%, respectively. The standard anti-inflammatory drug indomethacin similarly inhibited COX-1 and COX-2 by 82.8% and 54.6%, respectively. The IC_50_ values of EPMC for COX-1 and COX-2 were estimated to be 1.12 µM and 0.83 µM, respectively, whereas the corresponding values for indomethacin were found to be 0.33 µM and 0.51 µM, respectively.

### 2.2. Discussion

The rhizomes of *K. galanga* have been used in traditional medicine for the treatment of swelling for many centuries. Although a few preliminary investigations with the aqueous extract showed significant anti-inflammatory and analgesic effects [26,31], the constituents responsible for the anti-inflammatory effect of the herb were until this present study, not defined. Consequently, in the present study, *K. galanga* rhizomes were serially extracted with petroleum ether, chloroform, methanol and water in order to separate the constituents of the rhizomes according to their polarity. When these extracts were tested for anti-inflammatory effect, it was found that the % inhibition of inflammation by petroleum ether and chloroform extracts was significant, unlike the methanol and water extracts that did not exert significant effects when compared with control. Hence, the active anti-inflammatory agents in *K. galanga* were considered to be non-polar in nature or at the most, of intermediate polarity that can be dissolved in both petroleum ether and chloroform, but with a higher concentration in chloroform.

Fractionation of the chloroform extract yielded three fractions (F-1, F-2, and F-3). Upon testing for anti-inflammatory potential, F-3 was found to be the most effective (51.9% inhibition of inflammation). Unlike the chloroform extract, the effect of F-3 compared favorably with that of indomethacin, a standard anti-inflammatory agent. Accordingly, F-3 was further fractionated to obtain sub-fractions 1 and 2 (SF-1 and SF-2). Although both sub-fractions significantly inhibited inflammation, SF-1 was the most effective, with 53.8% inhibition of inflammation after 3rd h of carrageenan administration. Interestingly, the inflammation inhibition effect of SF-1 was found to be higher than that exerted by indomethacin (45.9%). This observation warranted a GC-MS analysis of SF-1 to indicate the purity and nature of compounds present in the sub-fraction.

A GC-MS analysis of SF-1 showed that it consisted of 80.05% of EPMC, 9% of β-sitosterol and 10.95% distributed amongst some other four trace components. The isolated crystals of EPMC from SF-1 when tested for inhibition of rat paw edema at doses of 100,200,400 and 800 mg/kg showed a potent dose-dependent anti-inflammatory effect with the minimum inhibitory concentration (MIC) of 100 mg/kg. It was further observed that the effect of EPMC at 800 mg/kg was not different from that of indomethacin, suggesting that EPMC could be the active anti-inflammatory constituent in *K. galanga* rhizomes, and may possibly share a similar mechanism of action with indomethacin. The development of edema is believed to be biphasic, with the first phase (first 1 h of carrageenan injection) caused by the release of histamine and bradykinin and an even more pronounced second phase (2nd to 3rd h) due to the release of prostaglandins [33,34]. Chloroform extract, F-3, SF-1 and pure EPMC inhibited both phases of carrageenan induced edema significantly which implies that the extracts and fractions may inhibit histamines, kinins as well as the prostaglandins to produce anti-inflammatory effect.

In an *in vitro* anti-inflammatory mechanistic study, EPMC was found to inhibit both COX-1 and COX-2 non-selectively. However, the inhibition of COX-2 was more pronounced (57.82%) compared to that of COX-1 (42.9%). Indomethacin is a non-selective COX inhibitor that exhibits its anti-inflammatory effect by inhibiting both COX-1 and COX-2. However, it is already documented that the inhibitory effect of indomethacin on COX-1 is comparatively more profound than its effect on COX-2. For instance, in a previous study, the IC_50_ of indomethacin was found to be 18 ± 3 nM and 26 ± 6 nM for COX-1 and COX-2 respectively [35]. Indomethacin showed the same trend in inhibiting COX-1 and COX-2 in our assay conditions with more profound inhibition of COX-1 (82.8%) than that of COX-2 (54.6%). Unlike COX-2, which is an inducible enzyme, COX-1 is constitutive, that is, present even in the absence of inflammatory conditions. In addition to the pro-inflammatory prostaglandins, COX-1 is responsible for the synthesis of those prostaglandins that are necessary for maintaining the integrity of gastro-intestinal mucosa. A higher inhibition of COX-1 increases the tendency of a drug to induce gastric ulcers and related complications. The observed inhibition of COX-2 by EPMC in this study, under the same conditions, was better than indomethacin, whereas the inhibition of COX-1 by EPMC (42.9%) was also far less than that by indomethacin (82.8%). This alternate action of EPMC when compared to indomethacin offers EPMC a comparative advantage over indomethacin, in treating inflammatory conditions particularly in patients with gastric ulcers. 

## 3. Experimental

### 3.1. Chemicals and Equipment

GC/MS-MSD: 6890N/5973 Agilent Technologies-Hewlett Packard model HP-5973 (Santa Clara, CA, USA), ^1^H-NMR Bruker 500 MHz Ultrashield (Billerica, Massachusetts, USA), cyclooxygenase inhibitor screening kits (Cayman Chemicals, Ann Arbor, MI, USA). Petroleum ether, chloroform, methanol, and *n*-hexane were obtained from Fisher Scientific (Selangor, Malaysia) and λ-carrageenan from Sigma-Aldrich, (St. Gallen, Switzerland).

### 3.2. Plant Material

Fresh rhizomes of *K. galanga* were collected from the Sungai Petani area of Kedah state, Malaysia in December, 2010, along with the vegetative parts. These were authenticated and a voucher specimen (voucher number 11251) preserved in the herbarium, School of Biological Sciences, Universiti Sains Malaysia, Malaysia.

#### 3.2.1. Extraction of Plant Material for Preliminary Activity Assessment

**Figure 6:**
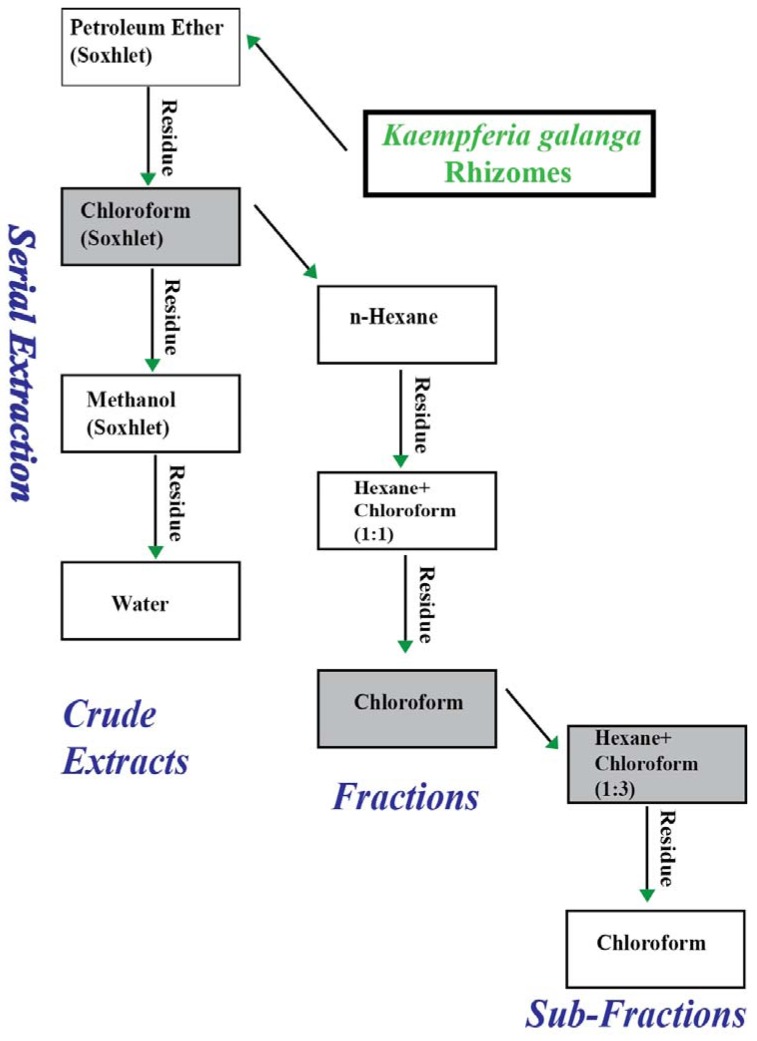
Schematic diagram showing the sequential extraction, fractionation and sub-fractionation of the dried rhizomes of *Kaempferia galanga.* Gray boxes represent the extract, fraction, and sub-fractions with maximum inhibition of rat paw edema.

The scheme used for the extraction of *K. galanga* is shown in Figure 6. The rhizomes were washed, chopped and dried in an oven at 45 °C. The dried rhizomes were powdered using a grinding mill. Three (3) kilograms of the dried powder of *K. galanga* was completely extracted, successively, in petroleum ether, chloroform and methanol using a Soxhlet apparatus. The residue left after methanol extraction was then extracted with distilled water by maceration at 45 °C overnight to obtain the water extract. The extracts were filtered, concentrated by using rotavapour, and thereafter freeze-dried. The freeze-dried extracts were then kept in the refrigerator at 4 °C until used.

#### 3.2.2. Liquid-Liquid Fractionation of Chloroform Extract

The chloroform extract was suspended in *n*-hexane and with gentle shaking; a brown coloured supernatant was formed. This layer was decanted and replaced with fresh hexane and repeated decanting until the supernatant became transparent (completely washed out). The collected fraction was filtered, concentrated using rotavapour and freeze dried to obtain fraction 1 (F-1). The residue was dried and then similarly washed with hexane-chloroform mixture (1:1) until no colour was formed with the solvent used. Again, this fraction was filtered, concentrated using rotavapour and freeze dried to obtain fraction 2 (F-2). Finally, the residue after the collection of F-2 was dissolved in chloroform, filtered, concentrated *in vacuo*, and freeze-dried to form fraction 3 (F-3).

#### 3.2.3. Liquid-liquid Fractionation of Fraction 3

Fraction 3 (F-3) was further extracted serially in hexane-chloroform mixture (1:3) and chloroform, using the same washing procedure explained in Section 3.2.2. This procedure yielded two sub-fractions, namely sub-fraction 1 (SF-1) and sub-fraction 2 (SF-2).

### 3.3. Chemical Analysis of the Most Effective Sub-Fraction (SF-1)

Qualitative analysis of the most effective sub-fraction was carried out using GC-MS under the following conditions: HP-5MS capillary column (30 m × 0.25 mm ID × 0.25 µm, film thickness); held at 70 °C for 2 min, raised to 285 °C at a rate of 20 °C/min and held for 20 min; 285 °C for MSD transfer line heater; carrier helium at a flow rate of 1.2 mL/min; 2:1 split ratio. 1 µL solution of SF-1 in chloroform (10 mg/mL) was injected automatically. Scan parameter low mass: 35 and higher mass: 550. The constituents were identified by comparison with standards using NIST 02. A total ion chromatogram (TIC) was used to compute the percentage of the identified constitutes.

Isolation of EPMC crystals from sub-fraction 1. SF-1 was taken in hexane in a conical flask and heated on a hot plate with drop wise addition of a 1:3 hexane-chloroform mixture from above till all the sub-fraction dissolved. The solution was allowed to cool at room temperature to get crystals of EPMC. The identity of the crystals was confirmed by ^1^H-NMR (*d_6_*-DMSO): 1.24 (3H, t, 1 × CH_3_, *J* = 12 Hz), 3.83(s, 3H), 4.60 (2H, q, 1 × CH_2_, *J* = 11.5 Hz), 6. 45 (1H, d, 1 × CH, *J* = 16.50 Hz), 6.97 (2H, d, 2 × CH, *J* = 14.5 Hz), 7.63 (3H, m, 3 × CH).

### 3.4. Pharmacological Procedures

#### 3.4.1. Animals

Male Sprague Dawely (SD) rats (150–200 gram) were obtained from the animal research and service Centre (ARSC), Universiti Sains Malaysia. The animals were kept in the animal transit room, School of Pharmaceutical Sciences, Universiti Sains Malaysia (23 °C temperature, 40–60% relative humidity, and 12 h dark/light cycle). The animals were provided free access to water and food *ad libitum*. However, the food was withdrawn 12 h before any experimental procedure was carried out on the animals. The experimental procedure and the use of animals received approval of the Animal Ethics Committee of Universiti Sains Malaysia (AECUSM) before the commencement of experiments [Reference number: PPSG/07 (A)/044/(2011) (63)/Approval number: USM/Animal Ethics Approval/2010/(63) (270)].

#### 3.4.2. Acute Toxicity Study in Rat

Healthy adult female SD rats (200–225 g) were used in the acute toxicity study. The study was conducted according to the fixed dose procedures (FDP; OECD guideline 4,202,001) [36]. The rats were divided randomly into five groups of six rats each. After an overnight fast, groups 1–4 were orally administered a single limit dose (5,000 mg/kg) each of petroleum ether, chloroform, methanol and water extracts of *K. galanga*. The extracts were reconstituted in 1% Tween 80 in distilled water; hence it was administered to group 5 which formed the control group. After this single dose extract administration, signs of possible toxicity were observed every hour for the first 6 h and every day for 14 days. All animals were weighed daily and observed for any signs or symptoms of toxicity and/or mortality for up to 14 days. Food and water consumption were recorded daily. The visual observations included changes in the skin and fur, eyes and mucous membranes, and also in the respiratory, circulatory, autonomic and central nervous system as well as somatomotor activity and behavioral pattern. A similar evaluation of EPMC toxicity was done later in the experiment. The animals were divided randomly into two groups of six rats each. Group 1 animals were orally given a single limit dose (2,000 mg/kg) of EPMC reconstituted in 1% tween 80 in distilled water. Group 2 animals were given 1% Tween 80 in distilled water. The animals were monitored for 14 days as mentioned above. In both acute toxicity studies, the animals were euthanized on the last day of experiment and LD_50_ value was estimated.

#### 3.4.3. Preparation of Test Samples and Dose Selection for Bioassays

A maximum dose of 2 g/kg was arbitrarily used for the preliminary assessment of anti-inflammatory effect of crude *K. galanga* extracts. However, after fractionation of the most active crude extract, the dose of the resultant fractions F-1, F-2 and F-3 used in the bioassay procedure was reduced by 50%, to 1 gram/kg. Likewise, the dose of the sub-fractions SF-1 and SF-2 was also reduced by 50%, to 500 mg/kg. EPMC isolated from the most effective sub-fraction was administered in graded doses of 100 mg/kg, 200 mg/kg, 400 mg/kg and 800 mg/kg. 1% Tween 80 in distilled water was used throughout the experiment to reconstitute the extracts. Indomethacin at 5 mg/kg (a dose simulated from human regimen) was used as reference drug at every stage of the experiment [37], while 1% tween 80 in distilled water was administered to the control group.

#### 3.4.4. *In Vivo* Anti-inflammatory Assay

Anti-inflammatory effect of *K. galanga* crude extracts, fractions, and sub-fractions was assessed by carrageenan-induced rat hind paw edema as described by Samud and co-workers [38] with minor modifications. Food was withdrawn from animals 12 h before the experiment; however they were given access to water *ad libitum*. Rats were divided into groups of six animals each (n = 6). *In vivo* assay of anti-inflammatory effect was carried out in four stages, *viz*: with the crude extracts (petroleum ether, chloroform, methanol and water extracts), fractions (fractions 1, 2 and 3), sub-fractions (sub-fractions 1 and 2) and isolated EPMC. At each stage, treatment groups consisted of rats given extracts/fraction/sub-fraction/EPMC, reference drug indomethacin (positive control) and 1% tween 80 in distilled water (negative control). Exactly 1 h after this oral treatment, 0.1 mL of 1% freshly prepared carrageenan in normal saline was injected into the sub-plantar region of right hind paw. The thickness of right hind paw of rats was then measured using micrometer before (baseline) and after 1st, 2nd and 3rd h of carrageenan administration. The % inflammation was calculated using following formula:


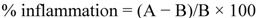
(1)

where A = Pad thickness, 3 h after carrageenan-induced edema; B = Pad thickness before carrageenan-induced edema.

#### 3.4.5. *In Vitro* Anti-inflammatory Assay of EPMC

Cyclooxygenase inhibitory effect of EPMC was assessed using cyclooxygenase (COX) inhibitory screening assay kits. Briefly, COX-1 (ovine) and COX-2 (human recombinant) were incubated separately with test samples (EPMC and indomethacin, each in a concentration of 200 µg/mL) for 15 min in test tubes and arachidonic acid was added. The reaction mixture was incubated at 37 °C in water bath for 2 min. This was followed by the addition of diluted hydrochloric acid and saturated stannous chloride solution into the reaction mixture of each test tube to stop the reaction. After an incubation of 18 h, the reaction mixture was taken on mouse anti-rabbit IgG coated micro-plate. Prostaglandin antiserum and later a prostaglandin tracer (prostaglandin antibodies linked with acetyl cholinesterase) were added to the wells of the micro-plate. After several washings, Ellman’s reagent was added to the wells and the absorbance of light was recorded in a micro plate reader at a wave length range of 405 to 420 nm.

### 3.5. Statistical Analysis

Data obtained from animal experiment were expressed as the mean ± standard error (±SEM). Statistical difference between the treatments and the control were evaluated by one-way analysis of variance (ANOVA) followed by Tukey’s multiple comparison test. Differences were considered significant at *p *< 0.05, *p *< 0.01, and *p *< 0.001.

## 4. Conclusions

Our results indicate that *K. galanga* possesses significant anti-inflammatory effect; hence the study contributes towards validation of the traditional use of *K. galanga* in the treatment of inflammation. Ethyl-*p*-methoxycinnamate isolated from *K. galanga *is found to be the vital anti-inflammatory constituent that exerts its anti-inflammatory effect via inhibition of the activities of cyclooxygenase enzymes 1 and 2.
